# The Ocular Glymphatic System—Current Understanding and Future Perspectives

**DOI:** 10.3390/ijms25115734

**Published:** 2024-05-24

**Authors:** Christine Delle, Xiaowei Wang, Maiken Nedergaard

**Affiliations:** 1 Center for Translational Neuromedicine, Faculty of Medical and Health Sciences, University of Copenhagen, Blegdamsvej 3B, 2200 Copenhagen N, Denmark; christine.delle@sund.ku.dk; 2 Center for Translational Neuromedicine, University of Rochester Medical Center, Elmwood Avenue 601, Rochester, NY 14642, USA; xiaowei.wang7@outlook.com; 3 Department of Ophthalmology, University of California, 10 Koret Way, San Francisco, CA 94117, USA

**Keywords:** glymphatic–lymphatic fluid transport, perivascular spaces, aquaporin-4, intraocular pressure, intracranial pressure, meningeal lymphatics, amyloid-beta, cerebrospinal fluid, blood–retinal barrier, glaucoma, diabetes

## Abstract

The ocular glymphatic system subserves the bidirectional polarized fluid transport in the optic nerve, whereby cerebrospinal fluid from the brain is directed along periarterial spaces towards the eye, and fluid from the retina is directed along perivenous spaces following upon its axonal transport across the glial lamina. Fluid homeostasis and waste removal are vital for retinal function, making the ocular glymphatic fluid pathway a potential route for targeted manipulation to combat blinding ocular diseases such as age-related macular degeneration, diabetic retinopathy, and glaucoma. Several lines of work investigating the bidirectional ocular glymphatic transport with varying methodologies have developed diverging mechanistic models, which has created some confusion about how ocular glymphatic transport should be defined. In this review, we provide a comprehensive summary of the current understanding of the ocular glymphatic system, aiming to address misconceptions and foster a cohesive understanding of the topic.

## 1. Ocular Non-Glymphatic Fluid Drainage Routes 

**Figure 1:**
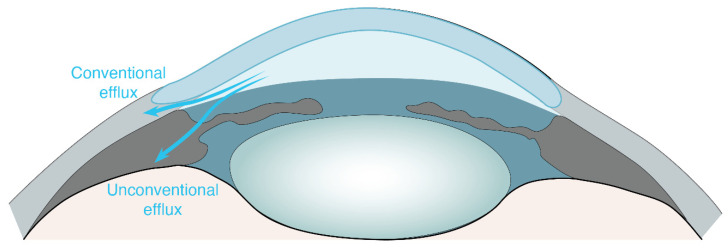
Simplified illustration of the traditional fluid efflux pathways in the anterior human eye. Aqueous humor leaves the anterior chamber either via the trabecular outflow pathway, also referred to as conventional pathway, or via the uveoscleral outflow pathway, also referred to as unconventional pathway.

Fluid homeostasis, which is vital for ocular function, has been a topic of intensive study in the anterior eye and the retinal pigment epithelium (RPE). The ciliary body, a specialized structure in the eye, produces the aqueous humor, which is of a similar composition to the cerebrospinal fluid (CSF) produced in the brain. The transparent aqueous humor provides optimal physiological conditions for light transmission through the eye. The aqueous humor also supplies the inner ocular structures with oxygen and nutrients and aids metabolic waste removal from avascular structures, such as the cornea and lens. The maintenance of physiological ocular pressure requires an equilibrium between aqueous humor secretion and its active removal. In a traditional view, two fluid drainage routes, the trabecular (conventional) and uveoscleral (unconventional) outflow pathway, are the critical mediators of anterior aqueous humor removal and the maintenance of ocular homeostasis [1,2,3] (Figure 1). 

The conventional pathway, which accounts for 70–95% of the aqueous humor outflow [4], entails the drainage of the aqueous humor through the trabecular meshwork into Schlemm’s canal and collector channels before entering the episcleral venous system [2,5,6]. In the uveoscleral outflow route, aqueous humor crosses over the iris root and through ciliary muscle tissue spaces and exits over several drainage routes, including efflux along supraciliary and suprachoroidal spaces, or via emissarial canals across the sclera and into choroidal blood vessels, likely passing along perivascular spaces [3,5,7,8,9]. In a later development, Yücel et al. (2009) described the ‘uveolymphatic outflow pathway’ in which ocular fluid drains from the anterior chamber via lymphatic vessels of the ciliary body into peripheral lymph nodes [10,11]. For an in-depth understanding of the mechanistic underpinning of the trabecular outflow pathway and uveoscleral outflow pathway, as well as a detailed illustration of the aqueous humor, we refer the reader to the outstanding reviews by Toris and Narayanaswamy et al. [3,12].

The ciliary double-layered epithelium facilitates water movement into the posterior chamber [13]. In the posterior eye, some fraction of the aqueous humor flows into the vitreous cavity, attaining the retina, followed by an efflux over the RPE [14]. The RPE cells form the outermost layer of the retina, bordering the photoreceptors on the apical side and the choriocapillaris on the basolateral side [15]. Among other important functions, RPE cells are particularly engaged in the regulation of retinal fluid homeostasis [16,17]. In the current model, RPE cells continuously remove fluid from the sub-retinal space [16,17,18], directing it towards the fenestrated and highly permeable choriocapillaris [19]. 

In addition to these pathways, Damasceno et al. recently reported the presence of lymphatic structures positive for the mucin-like transmembrane glycoprotein podoplanin in extraocular fat tissue, muscles, and the lacrimal gland in human ocular postmortem tissue, which could represent alternative ocular fluid drainage routes [20]. In that study, the authors also confirmed the presence of lymphatic structures in the optic nerve dura mater, in accordance with previous findings [21,22]. Recently, Yin et al. reported the presence of dural lymphatics around the murine optic nerve, positioned to drain solutes from the vitreous humor with a high specificity [23]. Such independent results confirm our previous observation that intravitreally administered human amyloid-β tracer drains along the dural lymphatic vessel in the rodent optic nerve [22].

## 2. Ocular Glymphatic Transport

Until the discovery of the brain glymphatic system, it was unknown how fluid homeostasis and metabolic waste removal are maintained in the brain. The brain glymphatic system, analogous to peripheral lymphatics, promotes a directed transport of interstitial fluid that clears water and metabolic wastes such as amyloid-β from the brain into the periphery [24]. From the subarachnoid space (SAS), CSF circulates along perivascular spaces which are plastered by astrocytic endfeet forming a semi-permeable barrier [25] that is permissive to water movement between the CSF compartment and the parenchymal interstitial fluid (ISF). Brain fluid flow is facilitated by the water channel aquaporin-4 (AQP4), such that its deletion (AQP4-knockout mice) exhibits a ~30–65% reduction in glymphatic CSF influx [24,26]. Further, the AQP4-dependent clearance of amyloid-beta from the brain was demonstrated [24,27]. Additionally, there is an AQP4-dependent clearance of tau protein, another aggregating protein involved in neurodegenerative diseases [28]. 

As the most metabolically active part of the eye [29,30], the retina is in constant need for neurotoxic waste removal. However, like the brain, the neuroretina, along with the optic nerve, lacks the typical lymphatic structures that are generally essential for the clearance of metabolic byproducts and excess fluid in other tissues. While there are efficient pathways for fluid efflux in the anterior eye, the basis for the fluid homeostasis of the anatomically complex and highly metabolic active retina seems less evident. While RPE cells are believed to facilitate fluid flow for the photoreceptor layer (the outermost retinal layer), the mechanism for metabolic waste removal and fluid homeostasis of inner retinal structures hosting the highly metabolically active retinal ganglion cells had long been uncertain. With the discovery of the brain glymphatic system [24], several members of the eye research community speculated that there must be an ocular glymphatic system [31,32,33,34,35,36]. 

The presence of fluid transport in the posterior eye is by no means a recent discovery, having been noted previously in various species, but the physiological importance of this process had been undefined [37,38,39,40,41,42,43]. Indeed, early electron microscopy studies reported the tracer exchange between CSF and ISF within the optic nerve [39,44,45,46]. In a 2016 study, Wostyn et al. substantiated the possible existence of an ocular glymphatic fluid route by tracking the perivascular distribution of India ink following postmortem subarachnoid injection in human optic nerves [47]. Shortly thereafter, Mathieu et al. demonstrated the existence of a glymphatic-like fluid transport from the brain to the optic nerve in mice, by tracking the distribution of an intracisternally injected tracer along perivascular spaces within the optic nerve [48]. Their localization of AQP4 along those perivascular spaces [48] paralleled the description of the brain glymphatic system [24,26].

In 2020, our group provided the first direct evidence for glymphatic fluid transport from the retina into the optic nerve in mice and rats [22]. We observed that intravitreally injected human amyloid-β (hAβ) and cadaverine tracers exited the retina via an intra-axonal route passing through the glial lamina to enter the optic nerve; the tracers continued along perivascular spaces and, finally, entered the perivenous space prior, ultimately leaving via the dural lymphatic vessels of the optic nerve [22]. In that report, we established for the first time that the ocular glymphatic system is a close analog of the brain glymphatic system by showing the following: (1) The perivascular expression of AQP4 is crucial for the ocular glymphatic system, as fluid transport is reduced in AQP4-knockout mice [22]. Of note, AQP4 expression was first demonstrated in the retina [49] and later described in proximity to perivascular spaces in the optic nerve [48]. (2) Fluid efflux from the retina occurs along the perivenous spaces, as with brain glymphatic efflux. (3) We confirmed that CSF enters the optic nerve and extended that observation to demonstrating that CSF flow occurs along the periarterial spaces [22]. Notably, we found that the ocular glymphatic system exhibits specialized characteristics that seem to fit its physiological circumstances: (1) Translaminar pressure, which is the difference between intraocular and intracranial pressures (IOP–ICP) per unit thickness of the lamina barrier, drives the fluid flow from the eye to the optic nerve. (2) The lamina between the retina and the optic nerve seems to behave as a selectively permeable barrier that allows some substances (e.g., amyloid-β and cadaverine) but not others (e.g., dextrans) to enter the nerve. (3) Light stimulation (1 Hz) promotes the inflow of fluid from the retina to the optic nerve. By pharmacologically inhibiting pupillary constriction, we showed that pupil constriction is likely the major driver of fluid transport in the optic nerve. (4) Intracisternally and intravitreally administered tracers are both drained into the optic nerve dural lymphatic vessels and continue into peripheral lymph nodes [22,50]. Importantly, we were able to confirm in our previous studies [22,50] the reports by Mathieu et al. documenting that intracisternally administered tracers were excluded from entering the eye under physiological conditions, as was more recently replicated independently [51].

Interestingly, AQP4 is not expressed in the rodent glial lamina or the lamina cribrosa in humans [49,52,53]. We speculate that the lack of AQP4 expression [52,53] aids to maintain the translaminar pressure gradient and restricts the fluid exchange between intraocular and intracranial fluid, which are vital aspects for maintaining fluid homeostasis by keeping IOP relatively isolated from physiological fluctuations in ICP. We conclude that the AQP4 dependence of the ocular glymphatic flow must be a consequence of the dense perivascular AQP4 expression in the optic nerve. 

Previous studies referred to the bidirectional glymphatic transport in the optic nerve as “ocular glymphatic transport”, regardless of the fluid source and distribution pathway [22,47,48,54]. However, it is critical that we distinguish the directions of fluid transport in the optic nerve, as the flow can originate from different sources, follow different routes, and depend on distinct biological mechanisms. The bidirectional aspect of the ocular glymphatic flow has raised some confusion in the past. For example, the recent report by Tong et al. (2024) confirmed prior studies reporting that intracisternally administered tracers do not enter the retina and that larger-molecular-weight dextran tracers travel shorter distances in the optic nerve [22,48,50,54]. Nevertheless, the Tong et al. study [51] inaccurately referred to both Mathieu’s and our findings by misquoting the studies as reporting that intracisternally injected tracers enter inner ocular structures. These statements are clearly confusing to the field: CSF tracers were never reported to pass through the glial lamina under physiological conditions. 

**Figure 2:**
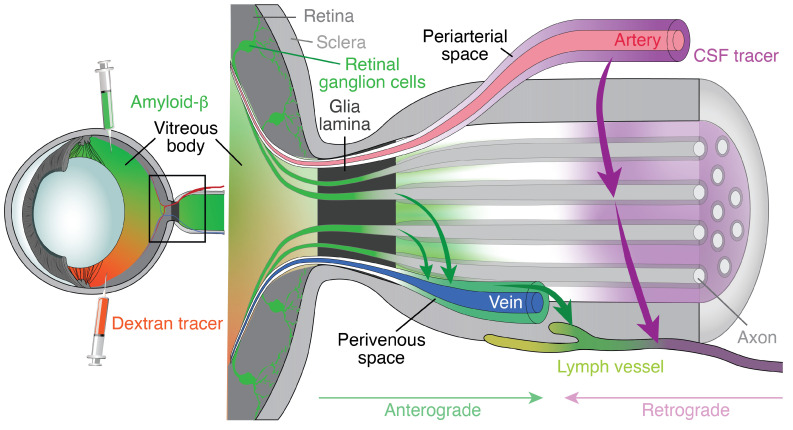
The ocular glymphatic system. The anterograde ocular glymphatic transport facilitates fluid and waste removal from the retina into the optic nerve. Left side: Injection of amyloid-β and dextran tracer into the vitreous. Right side: Detailed illustration of the black box area seen on the left. Intravitreal-injected amyloid-β (green) is taken up by the retinal ganglion cells and transported intra-axonally across the lamina barrier, whereas dextran tracers (orange) are excluded from the optic nerve and only enter the optic nerve if the lamina barrier is disrupted. In the optic nerve, amyloid-β is released by axons and accumulates along the perivenous spaces before being cleared by the dural lymphatic vessels. Retrograde ocular glymphatic transport denotes CSF transport that is transported into the optic nerve along the periarterial spaces. From here, CSF tracers (purple) efflux via dural lymphatic vessels and are also cleared along cervical lymphatic vessels. CSF tracers (dextrans and ovalbumin) can travel all the way to the lamina barrier but are excluded from crossing into inner ocular structures.

To distinguish the bidirectional ocular glymphatic transport pathways, we have recently designated fluid transport arising from the eye as ‘*anterograde ocular glymphatic transport*’, versus fluid arising from the brain and directed into perivenous spaces of the optic nerve as ‘*retrograde ocular glymphatic transport*’ [50,55]. Below, we display our current model of bidirectional ocular glymphatic transport in the optic nerve (Figure 2).

## 3. Ocular Glymphatic Transport in Aging and Ocular Diseases

Hitherto, studies of ocular glymphatic transport were conducted in rodent models in the settings of aging, glaucoma, and diabetic retinopathy [22,50,54,55]. We have observed that anterograde and retrograde ocular glymphatic transport both decline globally in aged mice [50], thus concurring with earlier reports of the brain glymphatic system impairing in aging [56,57]. While there is an earlier report of reduced retrograde ocular glymphatic transport in glaucomatous mice [54], our more recent study provided evidence for pathologically *increased* ocular glymphatic transport, along with enlarged perivascular spaces (PVS), in that model [50].

In a murine model of streptozotocin-induced diabetes, there were no discernible effects of chronic hyperglycemia on anterograde or retrograde ocular glymphatic transport [55]. Interestingly, in that same study, recurrent transient hyperglycemia induced by repeated glucose injections in otherwise healthy mice accelerated anterograde and retrograde ocular glymphatic transport. This increase in ocular glymphatic flow was associated with the dilation of the PVS in the optic nerve [55]. Despite the distinct underlying pathologies, it appears that increased ocular glymphatic transport in murine glaucoma and diabetic retinopathy models arises in relation to the altered PVS size [50,55]. As such, the PVS space enlargement in the optic nerve might reflect a pathologically increased ocular glymphatic flow, where the traffic of higher fluid volumes caused the PVS remodeling. However, there remains a need to identify the underlying pathological mechanisms in these phenomena. The ocular glymphatic pathway may well be the only anatomical conduit that allows communication between CSF and intraocular fluids. The recent study of Tong et al. in healthy rodents did not detect any entry of CSF tracers into the retina, leading the authors to question the existence of the ocular glymphatic pathway [51]. We here emphasize that Tong et al. misquoted prior publications; in fact, none of the earlier publications have reported that CSF tracers can pass through the glial lamina [22,48,50,54].

We shall next discuss the importance of using standardized techniques and terminology to ensure a coherent interpretation of the findings. 

## 4. Choice of Tracer to Study Ocular Glymphatic Transport

We believe it is important to note that the correct choice of tracer is of crucial importance for studying ocular glymphatic transport. Notably, hAβ and cadaverine tracers are thus far the only known tracers that are taken up by retinal ganglion cells and transported intra-axonally across the glial lamina into the optic nerve under physiological conditions [22]. Within an experimental time of 30 min, intravitreally injected dextran tracers did not discernibly cross the lamina barrier in healthy mice, but showed rapid and substantial entry into the optic nerve only when the barrier function was compromised, as in glaucomatous mice [22]. It is also crucial that we use fully fixable tracers, since tracer transport is frequently analyzed by the imaging of the ex vivo tracer distribution after harvesting the optic nerve. However, one needs to be aware that perfusion fixation can lead to tracer mislocalization and increased autofluorescence, which can bias the interpretation of results [26,58].

To study the glial lamina integrity, it is appropriate to intravitreally inject dextran tracers with differing molecular weights [22]. In murine glaucoma models, dextran tracers have been utilized to demonstrate the disruption of the glial lamina, which is visualized by the inflow of dextran tracers into the optic nerve [22]. As such, the administration of several dextrans across a range of molecular sizes can provide information regarding the spatial scale of the disruption in the lamina and the magnitude of the loss in the barrier function [22].

To investigate retrograde ocular glymphatic transport, the correct choice of tracer is also crucial. In earlier assessments of the distribution of CSF tracers, larger-molecular-weight dextran tracers did not travel the full length of the optic nerve [48]. In that study, high-molecular-weight (500 kDa) dextran failed to enter the optic nerve, while smaller dextrans (70, 40, and 10 kDa) did enter. Interestingly, the 70 kDa dextran had little access to the optic nerve (being detected only in one of four mice) [48], indicating that it could exceed a cutoff size for the sensitive detection of retrograde ocular glymphatic transport. Nevertheless, the study cohort was small (*n* = 4), and many experimental factors, such as variability in anesthesia or the body position of the animal, could have influenced the results. In some of our more recent studies, we utilized bovine serum albumin (BSA), which has a molecular weight of 66 kDa, and obtained sufficient glymphatic distribution in the optic nerve [50,55]. In the Mathieu et al. study, ovalbumin (45 kDa) proved to be an adequate tracer, with a comparable uptake to that of the similar-sized dextran (40 kDa) [48]. Tong et al. also confirmed the size-dependent distribution of intracisternally injected dextran tracers in the optic nerve, albeit for a restricted choice of sizes (2000, 70, and 10 kDa) [51]. From these studies, we conclude that the perivascular space of the murine optic nerve has size-exclusion properties, with an absolute cutoff around 500 kDa (dextran), and a yet unknown lower size limit. However, the cutoff seems to be close to 70 kDa, as the occasional glymphatic distribution was reported by Mathieu et al., and we noted, in two studies, that a 66 kDa tracer (BSA) was discernibly distributed via the glymphatic route in the optic nerve. Nevertheless, we see a need to study the distribution of distinctly smaller dextran tracers, such as 3 kDa, and to investigate their potential entry from the optic nerve into inner ocular structures. 

Findings that dextran or ovalbumin tracers of 45 kDa or a smaller size are suitable for studying the retrograde ocular glymphatic transport stand in agreement with the corresponding results for the brain glymphatic system [57,59,60,61,62]. We recommend the use of 10 kDa dextran for obtaining a deeper tissue distribution in retrograde ocular glymphatic studies [48,50,54,55]. In Table 1, we provide an overview of the different tracers used to study ocular glymphatic transport.

Tracers such as hAβ that are known to be transported along the glymphatic pathway are essential for studying the dependency of anterograde ocular glymphatic transport on AQP4 [22]. Tracers such as labeled tau proteins that emulate retinal metabolic wastes merit consideration in the future. Ocular diseases characterized by retinal amyloid deposits, such as age-related macular degeneration [63], may interact with an intravitreally administered hAβ tracer. Moreover, the contribution of amyloid generated in the retina calls for consideration. The ocular glymphatic system has not yet been studied in this context, but fluorescently labeled hAβ should enable a separation from the endogenous amyloid.

In contrast, due to their failure to track the anterograde glymphatic clearance from the retina, dextran tracers are not useful for studying the role of AQP4 in ocular glymphatic transport. Furthermore, as noted above, 10 kDa dextran tracers are fit to track the glymphatic pathway in periarterial spaces and should thus be considered for future studies of the AQP4-dependent retrograde transport [22,48,50,54]. On these grounds, we question the recent claim that glymphatic transport in the optic nerve does not depend on AQP4 [51]. In that study, the application of intracisternally administered 70 kDa in high volume and infusion rate (50 µL at 5 µL/min) may well have increased the intracranial pressure to an extent sufficient to force the larger-sized dextran into the optic nerve [51]. Furthermore, their assessment of the regional tracer signal in fixed optic nerve samples may have been vulnerable to artefacts arising from artificially induced intracranial pressure deviations. Therefore, we conclude that it is premature to conclude that retrograde ocular glymphatic transport occurs independently of AQP4 expression [51].

## 5. Importance of the Analytical Imaging Technique

When assessing ocular glymphatic transport, it is crucial to select the correct analysis technique to depict the differences between treatment groups and segmental changes of tracer accumulation, as argued above. This issue became especially prominent in the context of the increased glymphatic transport of the intracisternally injected tracer in the anterior part of the optic nerve of glaucomatous mice [50]. By an acute imaging of the entire length of freshly dissected glaucomatous optic nerves, we revealed a complex pattern of the CSF tracer distribution via the retrograde ocular glymphatic pathway [50], which was not evident in a previous study utilizing a cross-sectional analysis of the optic nerve [54]. Reporting the overall tracer signal along the entire optic nerve, the peak intensity, and the distance of the peak signal travelling from either the glial lamina for intravitreal-injected tracers or from the optic chiasm for intracisternally injected tracers can reveal new aspects of the bidirectional ocular glymphatic transport [22,50].

## 6. Importance of the Correct Injection Paradigm

Under physiological conditions, IOP exceeds ICP. Since the translaminar pressure difference (IOP–ICP) promotes directed fluid flow, the pressure difference at the glial lamina/lamina cribrosa might support glymphatic transport along the optic nerve from the retinal side (anterograde transport). Indeed, the manipulation of ICP by the infusion or withdrawal of CSF affected the anterograde ocular glymphatic fluid flow in mice; an ICP increase reduced the transport of the hAβ tracer from the retina into the optic nerve, whereas an ICP decrease significantly increased its transport into the optic nerve [22]. Notably, the IOP was unaffected during these ICP manipulations, remaining within the physiological range. Consequently, the ICP elevation could even abolish the otherwise increased anterograde ocular glymphatic transport during light stimulation [22]. 

As we noted above, Tong et al. tested the retrograde transport of dextran tracers upon an intracisternal injection of 50 µL at a flow rate of 5 µL/min [51], which would likely have provoked a significant ICP increase, consequently perturbing the glymphatic transport of the tracer along the optic nerve. While there is not yet any formal demonstration of such a phenomenon, non-uniform ICP increases might have caused a pressure gradient driving fluid (and tracer) into low-resistance pathways, as previously shown for the pial arterial PVS of the brain [64]. Indeed, the deeper and perivascular distribution seen in fixed optic nerve slices showed the strong penetration of the 70 kDa dextran tracer within the perivascular space [51], which scarcely occurs under more physiological conditions [48]. Thus, we urge caution in the interpretation of results obtained under non-physiological ICP conditions and in conjunction with optic nerve fixation. Interestingly, however, Tong et al. [51] may have provided insight into the effects of a high ICP on ocular glymphatic fluid transport and in potential fluid de-routing in pathological conditions such as hydrocephalus or stroke. 

In the same study, intracisternally injected dextran tracers travelled shorter distances along the optic nerve in response to the acute elevation of IOP in the range of 40–60 mmHg [51] previously reported in the optic nerve of glaucomatous mice with chronically elevated IOP [54]. In the scenario of retinal vein occlusion, which is characterized by disrupted blood flow to the retina and increased IOP, we expect that ocular glymphatic transport would be suppressed. Further, we also predict that retinal artery occlusion will negatively impact ocular glymphatic dynamics.

Furthermore, Tong et al. acutely deflated eyes during their intracisternal injection of dextran tracers, failing to detect the tracer signal within inner ocular structures such as the retina [51]. That negative result confirmed the result of previous studies reporting that CSF tracers do not pass through the glial barrier [22,48,50,54]. 

In consideration of artefacts likely arising from ICP perturbations, we have previously presented infusion protocols for intravitreally and intracisternally injected tracers having minimal effects on physiological pressure conditions, thus minimizing the possible impact on glymphatic distribution. For intravitreal tracer injections, we recommend delivering a total injection volume of 1 µL at a flow rate of 0.2 µL/min for no more than 5 min, and, for intracisternal tracer injections, we recommend delivering a total volume of 10–15 µL at a flow rate of 1.5–2 µL/min over a maximum of 10 min [22,50,55], as shown in Table 2. 

## 7. Future Directions

The ocular glymphatic system has so far been studied in rodents ex vivo [22,48,50,51,54,55], and in the human eye postmortem [47,65]. The currently utilized quantitative tracer analyses of acutely dissected whole optic nerves from rodents allow the depiction of fluid transport changes over the entire length of the optic nerve and provide an advantage over a segmental tracer analysis [51,54]. However, this method does not distinguish CSF tracer signals that are distributed along the SAS versus the perivascular route. To resolve this, one might undertake a detailed analysis of serial whole optic nerve cross-sections or sagittal sections with full reconstruction, or undertake the light sheet microscopy imaging of cleared whole optic nerves. We concede that such techniques would require optic nerve fixation prior to processing and analysis, which could cause bias due to the mislocalization of the tracer. Thus, studying the fluid flow in real time in a living organism would present a far more attractive experimental approach. However, currently available magnetic resonance imaging (MRI) strategies for small rodent models do not provide a sufficient spatial resolution to depict the perivascular fluid flow in the optic nerve. Larger-bodied animals such as pigs could be a more suitable model with which to study tracer-based imaging studies of the ocular glymphatic system. Nonetheless, barring substantial technical innovations, it shall remain technically challenging to study the glymphatic fluid transport within the optic nerve PVS of larger animals. Additionally, this approach comes with other challenges, as motion artefacts and signal spillover from surrounding tissue can impair the optic nerve in vivo imaging [66]. Moreover, we see a need for the development of novel tracers (such as an analog of amyloid-β) to study anterograde ocular glymphatic transport, as the currently available MRI tracers are not specific for the glymphatic pathway, but also label other ocular structures [67]. While awaiting further improvements of MRI or other modalities of non-invasive imaging, we propose that the best approach is to use a combination of techniques, while being mindful of their various limitations. 

When studying ocular diseases, it is relevant to assess both the anterograde and retrograde ocular glymphatic transport pathway. We note that little is known regarding the interactions of the bidirectional fluid transport within the optic nerve, i.e., how changes in one compartment influence the other. There may be compensatory mechanisms serving to maintain fluid homeostasis in the optic nerve. Moreover, compensatory mechanisms may not always be beneficial but might drive secondary impairments resulting in additional injury. For example, in the setting of ocular diseases such as glaucoma and diabetic retinopathy, the pathological dilation of the PVS in the optic nerve has been noted in conjunction with an increased glymphatic transport. Whether the increased fluid transport causes or is a consequence of PVS dilation is an important topic for future studies. It is of considerable interest to establish the prevalence of perivascular enlargement in blinding diseases of humans, and to understand better the underlying mechanisms that drive PVS size changes. By addressing such considerations, one might open a pathway to the targeted manipulation of ocular glymphatic fluid transport for the treatment of ocular diseases leading to blindness. 

## Figures and Tables

**Table 1:** Overview of different tracers used to study the ocular glymphatic system. Recommended tracers are labeled with *.

Tracer	Size (in kDa)	Reference	Suitability
**Study of retrograde ocular glymphatic transport (intracisternal injection)**			
Dextran *	2000	[51]	Unsuitable, too large
	500	[48]	Unsuitable, too large
	70 (*)	[48,51]	Low experimental experience; infrequent transport was reported [48]
	40 *	[48]	Suitable
	10 *	[22,48,50,54,55]	Suitable; optimal for deeper/farther tissue distribution
Ovalbumin *	45 *	[48]	Suitable
Bovine serum albumin	66 *	[50,55]	Suitable
**Study of anterograde ocular glymphatic transport (intravitreal injection)**			
Human beta-amyloid (1–40)	4.7 *	[22,50,55]	Suitable
**Study of glial lamina integrity (intravitreal injection)**			
Dextran	500 *	[22]	Usage of different-sized tracers aid to determine extent of barrier breakdown
	10 *	[22,55]	
	3 *	[22]	

**Table 2:** Recommended injection paradigms to study anterograde and retrograde ocular glymphatic transport.

**Anterograde ocular glymphatic transport—intravitreal injection**		
**Total volume**	**Flow rate**	**Total injection duration**
1 µL	0.2 µL/min	5 min
**Retrograde ocular glymphatic transport—intracisternal injection**		
**Total volume**	**Flow rate**	**Total injection duration**
10 µL	1.5 µL/min	10 min
15 µL	2 µL/min	10 min

## Abbreviations

AQP4	Aquaporin-4
CSF	Cerebrospinal fluid
ICP	Intracranial pressure
IOP	Intraocular pressure
ISF	Interstitial fluid
MRI	Magnetic resonance imaging
PVS	Perivascular spaces
RPE	Retinal pigment epithelium
SAS	Subarachnoid space
